# Nose-to-Brain: The Next Step for Stem Cell and Biomaterial Therapy in Neurological Disorders

**DOI:** 10.3390/cells11193095

**Published:** 2022-10-01

**Authors:** Natalia Villar-Gómez, Doddy Denise Ojeda-Hernandez, Eneritz López-Muguruza, Silvia García-Flores, Natalia Bonel-García, María Soledad Benito-Martín, Belen Selma-Calvo, Alejandro Arturo Canales-Aguirre, Juan Carlos Mateos-Díaz, Paloma Montero-Escribano, Jordi A. Matias-Guiu, Jorge Matías-Guiu, Ulises Gómez-Pinedo

**Affiliations:** 1 Laboratory of Neurobiology, Institute of Neurosciences, IdISSC, Hospital Clínico San Carlos, Universidad Complutense de Madrid, 28040 Madrid, Spain; 2 Preclinical Evaluation Unit, Medical and Pharmaceutical Biotechnology Unit, CIATEJ-CONACyT, Guadalajara 44270, Mexico; 3 Department of Industrial Biotechnology, CIATEJ-CONACyT, Zapopan 45019, Mexico; 4 Department of Neurology, Institute of Neurosciences, IdISSC, Hospital Clínico San Carlos, Universidad Complutense de Madrid, 28040 Madrid, Spain

**Keywords:** neurological disorder, remyelination, neurodegenerative disease, cell therapy, intranasal route, biomaterials

## Abstract

Neurological disorders are a leading cause of morbidity worldwide, giving rise to a growing need to develop treatments to revert their symptoms. This review highlights the great potential of recent advances in cell therapy for the treatment of neurological disorders. Through the administration of pluripotent or stem cells, this novel therapy may promote neuroprotection, neuroplasticity, and neuroregeneration in lesion areas. The review also addresses the administration of these therapeutic molecules by the intranasal route, a promising, non-conventional route that allows for direct access to the central nervous system without crossing the blood–brain barrier, avoiding potential adverse reactions and enabling the administration of large quantities of therapeutic molecules to the brain. Finally, we focus on the need to use biomaterials, which play an important role as nutrient carriers, scaffolds, and immune modulators in the administration of non-autologous cells. Little research has been conducted into the integration of biomaterials alongside intranasally administered cell therapy, a highly promising approach for the treatment of neurological disorders.

## 1. Introduction

Due to their severity, neurodegenerative diseases (ND) are an increasingly significant cause of morbidity worldwide [1]. While these diseases tend to be more prevalent in older age, they may also present at younger ages, and therefore affect patients of all ages [2]. NDs present heterogeneous etiologies and lack a clear genetic cause; nonetheless, a series of genetic mutations have been linked to several of these diseases [1]. Given the overlap of characteristics and symptoms between NDs and prion diseases, histology studies have shown that the protein aggregates identified in patients with NDs behave similarly to prions [3]. The available evidence suggests that these aggregates of misfolded proteins may migrate to other regions through cell–cell interactions and change the conformation of new structures due to a supposed selective vulnerability [4]. The appearance of protein aggregates is not limited to the later stages of disease; rather, they have also been observed prior to symptom onset [5]. NDs often involve demyelination, dendrite loss, neuronal death, neuroinflammation, and oxidative stress [2,6]. These disorders may be classified as amyloidosis, tauopathies, α-synucleinopathies, and TAR DNA-binding protein 43 (TDP-43) proteinopathies [5]. The proteins implicated in these disorders may constitute potential therapeutic targets and can be used as diagnostic biomarkers [5,7]. However, the majority of current treatments have focused on mitigating symptoms and slowing disease progression, and it is still necessary to develop targeted treatments to promote regenerative processes.

For many years, the growing incidence of NDs and the lack of functional treatments have motivated a search for new treatment strategies, such as the development of new drugs, cell therapy, and gene therapy. Cell therapy, in particular, has shown great therapeutic potential in NDs, due to the capacity of pluripotent or stem cells to promote tissue repair at lesion sites through the restoration of dysfunctional nerve cells and, consequently, to promote endogenous repair, neurorestoration, and neuroprotection mechanisms [8]. However, research on cell therapy for NDs is ongoing, and no definitive cure has yet been developed for any of these diseases. 

The blood–brain barrier (BBB) continues to represent a great challenge for the administration of treatments for NDs. Most therapeutic molecules are unable to cross the barrier to access the central nervous system (CNS) from the bloodstream; therefore, oral and systemic administration of these drugs is ineffective. Some alternatives have been proposed for the delivery of therapeutic molecules across the BBB, but most are only able to achieve low concentrations of these molecules [9]. Direct intracerebral or intrathecal administration to the CNS presents the disadvantage that it can be highly invasive, potentially triggering undesired responses [10]. Therefore, there is a need to develop biocompatible vehicles and unconventional administration routes to establish effective treatments targeting the CNS.

Intranasal administration offers a direct route to the brain, bypassing the BBB and avoiding potential adverse reactions associated with the systemic absorption of drugs. Compared to other administration routes, intranasal administration is non-invasive and highly practical, and may enable larger amounts of the therapeutic molecule to reach the brain [11].

This review discusses the advances made in the intranasal administration of cell therapies for each ND. We also address the advances in this field for the treatment of cerebrovascular disease (CVD), which, despite the different etiology from that of NDs, may also benefit from intranasal cell therapy. Finally, we present the potential use of biomaterials to assist in intranasal cell therapy targeting the CNS, a field that, to our knowledge, is as yet unexplored and may offer great therapeutic benefits in the treatment of NDs.

## 2. Intranasal Administration of Cell Therapy in the Treatment of Neurological Disorders 

**Figure 1:**
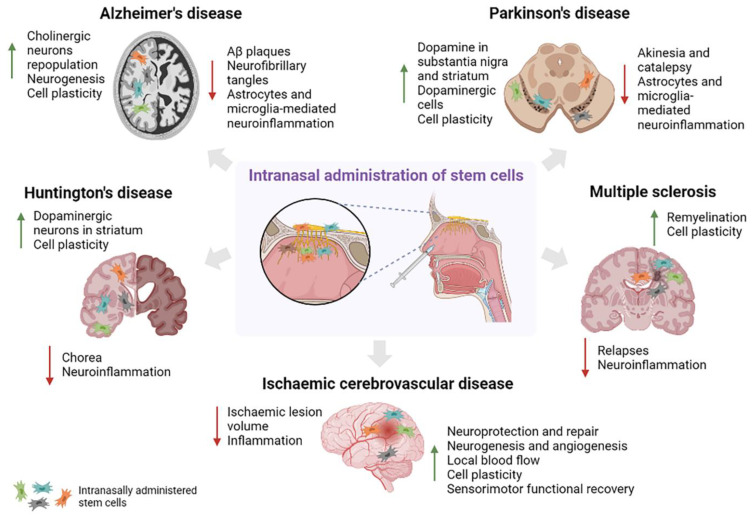
Expected effects of cell therapy administered via the intranasal route in the treatment of neurodegenerative and cerebrovascular diseases. The gray arrows indicate the possible therapeutic effects in the main pathologies.

One global effect of cell therapy, independent of the administration route, is that it promotes plasticity; for instance, it may be helpful in Alzheimer’s disease (AD), Parkinson’s disease (PD), amyotrophic lateral sclerosis (ALS), and stroke, as dendritic trees are able to cover and reconnect more brain areas [12]. Below, we describe the additional benefits reported in trials of intranasal cell therapy to treat ND and CVD (Figure 1).

### 2.1. Alzheimer’s Disease

AD is the most frequent ND and the leading cause of dementia. It is a disease of multifactorial aetiology but is mainly associated with the accumulation of β-amyloid (Aβ) and intracellular neurofibrillary tangles as a result of tau hyperphosphorylation and neuronal death [6,13,14]. The four medications currently approved by the United States Food and Drug Administration for the treatment of AD target the symptoms of the disease, comprising acetylcholinesterase inhibitors and an N-methyl D-aspartate receptor antagonist [15].

In the context of AD, cell therapy aims to repopulate acetylcholinergic neurons with a view to restoring neuronal circuits [14]. In 2014, Danielyan et al. [16] intranasally administered macrophages to APP/PS1 mice; the cells reached several brain areas, including the hippocampus and occipital cortex. Furthermore, the macrophages identified in these regions were located close to Aβ plaques, and some were Aβ-positive, indicating a process of phagocytosis [16]. In a more recent study, intranasal administration of human neural stem cells (NSC) resulted in a decrease in the size and number of Aβ plaques in the hippocampus and cortex, and a reduction in the number of astrocytes and microglia. Furthermore, these cells were able to differentiate into cholinergic neurons after reaching the cortex. These authors also detected increased levels of the marker doublecorxin (DCX) in the subgranular zone of the dentate gyrus after human NSC transplantation, suggesting increased neurogenesis. Finally, animals receiving the cell treatment presented improvements in learning and memory [17]. 

### 2.2. Parkinson’s Disease

PD is the second most frequent ND and the most common form of parkinsonism [3,18]. The disease is characterised by a progressive loss of dopaminergic neurons in the substantia nigra pars compacta and the appearance of Lewy bodies, insoluble protein aggregates mainly composed of α-synuclein [18,19,20]. Currently, pharmacological treatments are used to control motor symptoms of PD, aiming to improve dopaminergic signaling by direct replacement (with levodopa), dopamine receptor agonism, and the reduction of dopamine metabolism by inhibiting monoamine oxidase B and catechol-O-methyltransferase [3].

In PD, cell therapy approaches mainly aim to regenerate dopaminergic neurons [21]. Potential treatment strategies in cell therapy involve increasing the use of induced pluripotent stem cells or induced neurons derived from fibroblasts from patients with PD, enabling reversion of the main symptoms of PD and better evaluation of the effects of medications in preventing neurodegeneration [20,22]. However, special attention must be paid to the cocktail of transcription factors used to reprogram fibroblasts due to their tumorigenic potential [20].

PD is the ND for which the most trials have been conducted on the use of the intranasal pathway for cell therapy. The first study was conducted in 2011 by Danielyan et al. [23], who administered mesenchymal stem cells (MSC) in the 6-hydroxydopamine (6-OHDA) rat model of PD. That study demonstrated that the cells were able to survive for up to 4.5 or 6.7 months after administration and proliferated at the lesion site. The authors also observed increased levels of dopamine and of the marker tyrosine hydroxylase (TH) in the damaged tissue of the striatum and substantia nigra. Intranasal administration improved the function of the forelimbs [23]. These results raised interest in the use of the intranasal route for the treatment of PD. A year later, the same rat model was used in a trial of human MSCs, which were tagged with a fluorescent marker for tracing with near-infrared imaging, enabling non-invasive analysis of the distribution of the implanted cells in vivo [24]. 

In diseases like PD, whose pathophysiology is not fully replicated by the available animal models, it is important to use a range of models to confirm the efficacy of potential treatments. Intranasal administration of MSCs in the (Thy)-h[A30P]αS transgenic mouse model resulted in the migration of these cells to the CNS, and particularly to the olfactory bulb and brainstem, in close proximity to phosphorylated α-synuclein aggregates [16]. In another study, NSCs were transplanted into MPTP mice, resulting in behavioral improvements, mitigation of the loss of dopaminergic neurons in the substantia nigra pars compacta, and decreased inflammation secondary to astrocytic and microglial activation. The same study also showed that these effects were enhanced when NSCs were combined with the drug fasudil [25]. In another study, in which dental pulp stem cells were administered in the same animal model, researchers observed an improvement in sensorimotor coordination and increased TH labeling, even 4 weeks after administration [26]. 

In 2017, Salama et al. [27] studied the intranasal administration of MSCs in the rotenone model, observing an increase in dopaminergic neurons in the substantia nigra pars compacta and fibers in the striatum. Locomotor functions affected by rotenone administration were restored with cell transplantation, with a reduction in akinesia and catalepsy [27]. A subsequent study used human endometrial-derived stem cells. Intranasal administration of these cells in the 6-OHDA model led to an increased number of dopaminergic neurons in the substantia nigra pars compacta. Cell therapy also improved the animals’ motor behavior. The authors observed a dose-dependent effect, with the optimal dose being 5 × 10^4^ cells/µL^–1^ [28]. Finally, a recent study used olfactory ecto-MSCs. Intranasal administration of these cells in the 6-OHDA rat model increased the levels of dopaminergic markers and improved performance in behavioral tasks [29]. 

### 2.3. Huntington’s Disease

Huntington’s disease is a genetic neurodegenerative disease in the group of polyglutaminopathies. The disease is caused by excessive repeats of the CAG triplet in the *HTT* gene, encoding the protein huntingtin. The mutation results in a truncated protein with a toxic effect, losing its physiological function. The clinical trials currently underway focus mainly on eliminating the truncated copy of *HTT* using RNA interference techniques or antisense oligonucleotides [30].

Cell therapy approaches to Huntington’s disease mainly aim to regenerate striatal neurons [30]. In one recent study, MSCs were administered to the R6/2 mouse model, via both the intranasal and intravenous routes. Both routes were viable, improving the animals’ motor behaviour, restoring circadian rhythms, and reducing levels of inflammatory mediators in the olfactory bulb, hippocampus, and striatum. Furthermore, the administration of MSCs was associated with an increase in levels of DARPP-32 and TH, which are markers of mature medium spiny neurons and dopaminergic neurons, respectively. Another interesting finding is that the cells administered intranasally presented higher survival rates at 11 weeks [31]. 

### 2.4. Multiple Sclerosis

Multiple sclerosis (MS) is a chronic autoimmune disease of the CNS, considered the most common disabling disease in young adults [32,33]. It is characterised by inflammation, demyelination, large focal lesions to the white and grey matter, and axonal loss. As is the case with most other NDs, MS etiology is multifactorial, with both genetic and environmental factors playing a role. Treatment of MS is centred around the use of disease-modifying treatments that target the inflammatory component, reducing the number of relapses. However, these therapies are unable to revert the axonal damage that occurs at progressive stages [33,34].

In MS, cell therapy aims to promote functional recovery through myelin regeneration or remyelination. Numerous types of stem cells have been used for that purpose, with human MSCs and NSCs being the most frequent [35]. Few studies to date have used intranasal delivery of cells in animal models of MS, with the majority using the experimental autoimmune encephalomyelitis (EAE) model. In 2014, Fransson et al. [36] used modified MSCs expressing a specific receptor for myelin oligodendrocyte glycoprotein. Cells were administered intranasally in EAE mice, reaching areas including the olfactory bulb, entorhinal cortex, and cerebellum. This resulted in reduced inflammation and demyelination, as well as clinical improvement [36]. In another study published the same year, mouse adult NSCs were delivered intranasally and intravenously to EAE mice, and their distribution was studied at 1, 7, and 21 days after transplantation. On day 1, cells reached areas including the olfactory bulb, cortex, hippocampus, striatum, and brainstem. However, they were not detected in the spinal cord. By day 7, the cells administered intranasally were found in the spinal cord; by day 21, large numbers of cells administered by both routes were found in demyelinated areas of the white matter. Administration of adult NSCs had an anti-inflammatory effect that was restricted to the CNS and did not affect the peripheral immune response. The authors also observed increased remyelination in the ventral column of the lumbar spinal cord. After reaching the CNS, cells were able to differentiate into NG2^+^ oligodendrocyte precursor cells (OPC), mature oligodendrocytes, astrocytes, and neurons [37]. Gómez-Pinedo et al. [38] used the intranasal route to administer oligodendrocyte-lineage cells, which reached the olfactory bulb and migrated to areas with quiescent endogenous OPCs (fimbria, corpus callosum, and septum); this was one of the first studies to use this cell lineage for intranasal administration.

### 2.5. Amyotrophic Lateral Sclerosis

ALS is a neurodegenerative disorder characterized by progressive loss of upper and lower motor neurons in such areas as the motor cortex, brainstem, and spinal cord. The disease is lethal, with an average life expectancy of approximately 5 years after diagnosis. Currently, only two drugs are used to treat ALS, although neither prevents disease progression [39]. Research is being conducted into new treatment approaches, such as gene editing and stem cell transplantation. 

Stem cell treatments are mainly being used to promote the migration of neurotrophic factors to the lesion site to stop the degenerative process [39,40]. However, to our knowledge, the intranasal route has not yet been used to administer these treatments.

### 2.6. Ischemic Cerebrovascular Disease 

A general discussion follows of the literature on the intranasal administration of cell therapy in ischemic CVD, with a particular focus on neonatal hypoxic-ischemic (HI) brain injury and ischemic stroke. For greater detail on studies into the intranasal administration of cell therapies in the treatment of HI brain injury, we recommend the recent review by Salahi et al. [41].

### 2.7. Neonatal Hypoxic-Ischemic Brain Injury

Neonatal HI brain injury presents in 2.9 of every 1000 newborns and is one of the main causes of death in this population group; it also causes long-term neurodevelopmental problems. Currently, the only approved treatment is therapeutic hypothermia. However, this is only efficacious in full-term newborns, with a success rate of 45%. The other treatment options only focus on post-treatment, controlling such symptoms as hypoglycemia, seizures, etc. In this context, the need arises to search for other alternatives, such as the use of cell therapy to repopulate or regenerate the damaged area of the brain [42,43].

The intranasal administration of cells to treat neonatal HI brain injury was first studied by Donega et al. [44] in 2013. These researchers induced HI injury in 9-day-old mice by permanent occlusion of the carotid artery. Bone marrow-derived MSCs (BMSC) were administered intranasally 3, 10, or 17 days after the injury, to determine the therapeutic window. Treatment with the cells was associated with decreased loss of grey and white matter at 5 weeks after HI injury. When they were administered 3 days after HI injury, they reached the lesion site in the hippocampus, and at 10 days they spread to damaged cortical areas. However, at 17 days, the treatment had no effect. The authors also studied the possibility of administering multiple doses, finding no differences compared to the use of a single dose. The group that received the cells also presented improvements in sensorimotor and cognitive behavior [44]. Two years later, the same research group studied the long-term effects of the same procedure, finding that the benefits of treatment persisted for up to 14 months after HI injury. The study also included a comprehensive analysis of the appearance of malformations or tumors in the nasal turbinates, brain, and peripheral organs to verify the safety of administering BMSCs [45]. In another study from 2015, the same type of cell was administered to rats 6 h after stroke, resulting in reduced infarct volume and BBB protection. Seventeen days after the HI injury, the authors observed the expression of markers associated with neurogenesis and angiogenesis, as well as improvements in local blood supply and in sensorimotor, olfactory, and social function [46].

### 2.8. Ischemic Stroke

Ischemic stroke is the third leading cause of death worldwide, and the leading cause among women. Ischemic stroke is a type of CVD in which the severity and duration of ischemia may lead to the appearance of neurological symptoms over time [47]. Ischemic stroke is currently treated with a tissue plasminogen activator, which catalyzes the conversion of plasminogen into plasmin, dissolving fibrin and, consequently, the blood clot. On the other hand, new treatments under development seek to favor neuroprotection and neuroregeneration after stroke; cell therapy is one of the strategies being explored in this context [48]. To that end, some researchers have evaluated the administration of stem cells in animal models with a view to regenerating the ischemic brain tissue. NSCs and embryonic stem cells have the potential to differentiate into neural lineages after occlusion of the middle cerebral artery, in light of the evidence that these cells can give rise to neurons at the lesion site [49]. 

In the context of ischemic stroke, the intranasal route was first used by Wei et al. [50] in 2013. This study used the middle cerebral artery occlusion model in mice, with BMSCs administered 24 h after stroke. Cells reached the CNS 1.5 h after transplantation, and hypoxic-preconditioned cells were mainly concentrated in ischemic areas. The authors also observed decreased infarct volume 3 days after administration, with reduced cell death and improved sensorimotor function [50]. In 2021, the same research group implanted BMSCs together with insulin-like growth factor 1 at 3, 5, and 7 days after stroke. Two weeks after stroke, the animals treated with BMSCs presented increased expression of NeuN and Glut-1, which are markers of neurogenesis and angiogenesis, respectively. This was confirmed with the detection of increased expression of the proteins BDNF, VEGF, and Ang-1, which are also involved in these processes. Cell therapy also improved local blood flow and motor function [51]. 

## 3. Transport through the Nasal Cavity and Distribution of Stem Cells

Intranasally administered molecules and substances reach the brain parenchyma via the olfactory or trigeminal nerves. They are then able to spread throughout the brain. This process may occur by either an intracellular or an extracellular pathway. While the intracellular mechanism consists of the endocytosis and transportation of the molecule by olfactory neurons, the extracellular pathway is more complex, as the molecule must cross the olfactory epithelium to the lamina propria, where it is subsequently transported along the neuronal axon, directing the molecule to the CNS [52]. A better understanding of the permeability characteristics of the olfactory epithelium and the mechanisms involved in the transport of substances from the epithelial surface to the lamina propria will enable the selection and design of better intranasal treatments for NDs [53].

In the case of intranasal cell therapy, it is essential to confirm that stem cells successfully reach the CNS after administration [54]. While some studies report that intranasally administered MSCs labeled with enhanced green fluorescent protein do not survive or migrate to the brain [55], other authors observed that a large number of stem cells enter the brain and accumulate specifically at lesion sites, with effects lasting for months [54]. For instance, NSCs reached an area of intracerebral glioma within 6 h of intranasal administration and increased considerably in number within 24 h. The majority of NSCs were enriched in the tumor area, with only a few transplanted cells being observed in the hippocampus and subventricular zone [56]. Following intranasal administration, MSCs have also been detected in the olfactory bulb, brain, and spinal cord, with approximately a quarter of cells surviving for at least 4.5 months in the brain in a rat model of PD [23]. However, further research is needed into the migration, distribution, and survival of stem cells in the CNS after intranasal administration in the context of each disease.

Table 1 below shows the main cell types used in intranasal injection, where limitations and strengths are described.

## 4. Limitations of Nose-to-Brain Cell Therapy

Despite the success of various clinical trials using stem-cell-based therapies, research is also being conducted to test different strategies to achieve greater efficacy in the release of these cells and their function in the target organs [54] as not all cells reach the brain parenchyma following intranasal administration. Galeano et al. [57] demonstrated that only a small quantity of intranasally administered MSCs entered the CNS across the cribriform plate, with the majority entering the space below the turbinate bones [57]. In addition to this, the distribution of drugs or cells after intranasal injection varies according to the animal model used. Even in experiments using the same animal model, the efficacy of intranasal administration varies according to the age and weight of the animal [58]. Krishnan et al. [59] injected radiolabeled pralidoxime into the nasal cavity of rats, finding that compared to younger animals, older animals with greater body weight required higher doses to reach the same concentration of the drug in the brain. 

In this regard, stem cells have been genetically engineered to overexpress genes promoting survival or differentiation [60] and cytokines [61]. The co-administration of clinically proven drugs (neuroprotective, antiapoptotic, antioxidant, or immunomodulatory agents) and stem cells has been shown to considerably improve the efficiency of implantation and the survival of the cells, enhancing their therapeutic potential in the target organ, in comparison with stem cells alone [62]. Hyaluronidase may also be administered prior to stem cells to reduce the barrier function of the nasopharyngeal mucosa, improving their distribution to the brain [63]. Transplant efficiency may also be affected by small technical details. All of these experimental considerations are discussed in the review by Dhuria et al. [64] and include the position of the subject’s head during administration, the volume of the injected cell-containing vehicle, and the administration technique (injection into one or both nostrils; use of a tube, pipette, dropper, etc.).

The existent studies show how drugs reach the CNS whereas little information is available for cells. It is believed that the mechanisms involved can be the same, being these the following [64]:

Olfactory nerve pathway: it is considered the most predominant one since there is a positive correlation between the concentration of substances in the olfactory epithelium and olfactory bulbs.

The olfactory epithelium is composed of olfactory receptor neurons (ORNs), supporting or sustentacular cells, and basal cells. The ORNs have dendrites that contact the odorants and axons that cross the lamina propia and form nerves. These nerves cross the cribriform plate and reach the glomerulus of olfactory bulbs.

This transport can be classified into:

- Intracellular: cells get through the ORNs via passive diffusion or endocytosis. This pathway requires more time than the extracellular one as endocytosis is a slower mechanism.
- Extracellular: cells reach the olfactory bulb through the spaces between the tight junctions that connect the ORNs and sustentacular cells.

Trigeminal nerve pathway: the trigeminal nerve innervates both the respiratory and olfactory epithelium. Cells enter the CNS by reaching the olfactory bulbs (rostral) or the brainstem (caudal). 

Vascular pathways: it is believed that this type of transport happens because of the absorption of substances by the capillaries beside the nasal mucosa. The olfactory mucosa has several blood vessels, and this transport is suitable for small and lipophilic molecules. There are several possibilities for vascular pathways:

- Systemic circulation: substances get systemic circulation through the fenestrations in the vasculature and then must cross the BBB to reach the CNS.
- Venous blood: substances can be transferred to the carotid arteria that reaches the brain and spinal cold.
- Perivascular spaces: perivascular spaces are like channels that surround the blood vessels, and they act as a drainage system. These spaces clear the substances that come from the neurons.

Cerebrospinal fluid pathways: the substances can reach the CSF directly and be distributed to the brain and spinal cord.

**Figure 2:**
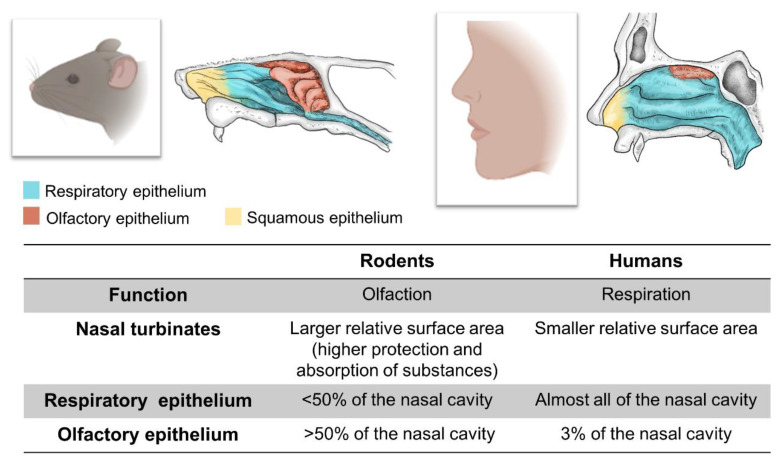
Comparison of the nasal atrium in rodents and humans, presenting the differentiating characteristics that may affect the efficacy of cell delivery to the CNS.

Another crucial aspect to be taken into account when transferring advances in intranasal cell therapy to the clinical setting is the differences between rodent and human olfactory systems. Firstly, the rodent’s olfactory system is far more complex than that of humans, with the system’s primary function being olfaction in rodents and respiration in humans. Thus, the surface area of the nasal turbinates is comparatively greater in rodents than in humans, enabling better protection of the lower respiratory tract and greater filtration and absorption of substances. Most animal species present four epithelia: the squamous, respiratory, transitional, and olfactory epithelia. The most noteworthy differences between rodents and humans are observed in the respiratory and olfactory epithelia. In humans, the respiratory epithelium covers almost the entire nasal cavity, compared to less than 50% in rodents. Similarly, the olfactory epithelium covers only 3% of the nasal cavity in humans, compared to 50% in rodents. These anatomical differences may influence the efficacy of intranasal cell transplantation, as the most widely accepted hypothesis regarding the mechanism of this administration route is that cells reach the CNS by crossing the olfactory epithelium [65,66] (Figure 2). 

Despite these limitations, several clinical trials have been conducted to test intranasal therapies for neurological disorders. For instance, BMSCs have been used to treat AD, cognitive disorders, and other NDs, whereas NSCs have been administered to patients with PD to assess their safety and efficacy (ClinicalTrials.gov; Identifiers: NCT03724136, NCT02795052, NCT03128450). Details of clinical trials conducted to date are shown in Supplementary Material Table S1.

## 5. Biomaterials in Nose-to-Brain Therapies

In addition to the aforementioned limitations of the intranasal administration of cells, and despite the promising results of these treatments for ND and CVD, cell therapy may be insufficient in the case of very extensive lesions. This may be because stem cells are unable to migrate to the lesion site, or because they are unable to survive or differentiate into functional cells as they require a support-providing structure and a favorable microenvironment for repairing damage [67,68]. In this respect, biomaterials have been proposed as a tool to promote a microenvironment favoring regeneration in the context of ND.

The biomaterials currently used include natural materials such as chitosan, collagen, and hyaluronic acid, due to their high levels of biocompatibility and biodegradability; however, it is often necessary to modify their chemical structure or to combine them with synthetic biomaterials to achieve specific mechanical properties [69,70,71]. Through these modifications, it is also possible to achieve new biological properties and to develop final products with a certain versatility, enabling different presentations to fit the needs of a specific application [72]. As a result, biomaterials have been used in cell therapy to mimic specific aspects of CNS tissues to provide cells with structural support or protection, increasing their effectiveness [73]. In biomaterials used for structural support, biocompatibility and biodegradability play a key role, as the materials must be progressively replaced by regenerated material, without generating potentially toxic products [74,75]. Ghuman and Modo [76] propose that, moreover, the ideal biomaterial must protect the implanted cells from the host response and interact positively with the immune response. In this way, the incorporation of different biomaterials in cell therapy targeting the CNS has been shown to promote cell survival, integration, and differentiation [77].

**Figure 3:**
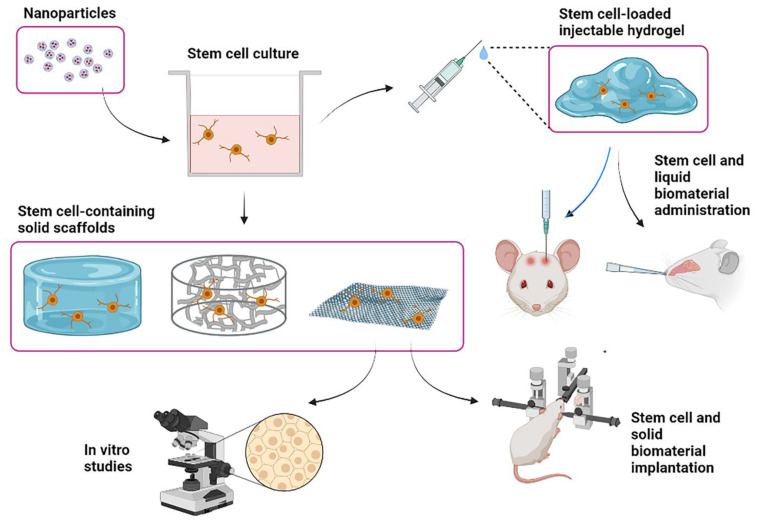
In vitro and in vivo uses of biomaterials in research and for cell therapies to promote regeneration in the context of neurological disorders. Applications include the addition of nanoparticles to the culture medium to guide the behavior of stem cells, the use of solid supports (e.g., hydrogels, nanofibrous scaffolds, and graphene) containing stem cells for in vitro studies and for intracerebral implantation in animal models, and the use of injectable hydrogels for intracerebral or (in the future) intranasal administration.

The administration of therapeutic molecules targeting the CNS can also be assisted with the use of biomaterials. This is mainly due to their role as carriers, protecting the molecules from degradation and conferring new characteristics to improve their stability and effectiveness [70]. For instance, nanocarriers have been used to facilitate the passage of drugs across the BBB and to enable controlled release, with highly specific interaction at the molecular level [78]. Similarly, biomaterials have been used to facilitate the intranasal administration of drugs; in this context, stimulus-responsive hydrogels have gained special importance due to their ability to promote better distribution of the molecules across the brain [79]. Biomaterial applications seek to overcome some of the obstacles of nose-to-brain treatments: the small surface area of the human nasal cavity, which limits the volume that may be administered; the lipophilicity of the mucosa, which hinders the administration of hydrophilic molecules; and mucociliary clearance, which reduces retention time in the nasal cavity [79]. For example, it is possible with formulations containing mucoadhesive biopolymers to increase and control the retention time of the drug in the nasal cavity, facilitating its passage to the brain [80,81]. The use of thermosensitive gels that are administered as a liquid and subsequently undergo gelation in the nasal cavity prevents the formulation from dissipating outside the nose or flowing towards the throat [82]. It is also possible to design amphiphilic biomaterials, such as some micro- and nanocarriers, to facilitate the intranasal administration of hydrophilic molecules [83] (Figure 3).

Below, we present some of the benefits of biomaterials, both for cell therapy and for other therapeutic molecules, in the nose-to-brain administration of treatments for NDs and CVD.

### 5.1. In Vitro Studies

The use of biomaterials in cell cultures can guide stem cells towards a specific differentiation. The most extensively studied biomaterials for this purpose are nanofibrous scaffolds produced with synthetic compounds, which are sometimes also mixed with natural compounds. For example, nanofibrous scaffolds of poly-L-lactic acid (PLLA) copolymerized with poly(ε-caprolactone) (PCL) and collagen have been used to guide MSCs to neuronal differentiation [84]. A similar response was observed with MSC cultures using copolymeric nanofibrous meshes prepared with amine-terminated poly(ethylene glycol) conjugated to PCL and functionalized with a nerve growth factor applied to the amine surface [85]. Cell cultures on PLLA nanofibrous scaffolds have also been used to guide the differentiation of MSCs from the trabecular meshwork of the eye into dopaminergic neurons [86]. Similarly, Matrigel-coated nanofibrous PCL scaffolds have been used to culture Nurr1/GPX-1–expressing embryonic stem cells, providing support and promoting the functional generation of dopamine-secreting cells, a finding with great potential in cell therapy for PD [87]. The combination of PLLA with gelatin has been used to develop three-dimensional nanofibrous scaffolds that increase the differentiation of induced pluripotent stem cells to neural-like cells [88]. Another study found that PLLA nanofibrous scaffolds promote the adhesion, growth, survival, and differentiation of NSCs derived from induced pluripotent stem cells. The authors also observed that aligned PLLA nanofibers directed and promoted neurite outgrowth, compared to randomly oriented fibers, demonstrating the importance of biomaterial microarchitecture [89]. Another study showed that the initiation of processes related to remyelination by OPCs or by oligodendrocytes may be sensitive to the diameter of poly-(L-lysine)–coated nanofibers [90]. 

Studies using graphene have shown that, compared to two-dimensional biomaterials, three-dimensional structures promote greater NSC and PC12 growth and cause less neuroinflammation, demonstrating the importance of the structural topography of biomaterials [91]. Graphene biomaterial surface topography has also been found to affect the adhesion and guidance of neurites [92]. Studies with three-dimensional graphene scaffolds suggest that greater rigidity may favor the differentiation of NSCs to astrocytes, demonstrating the importance of the flexibility of the cellular environment [93]. While graphene enables the design of very precise microstructures, very promising results have been reported with the combination of graphene and natural biomaterials. For instance, in a study using a three-dimensional bacterial cellulose–graphene foam, the biomaterial not only favored the adhesion and growth of NSCs but also helped to maintain stemness and to improve proliferative capacity. Furthermore, it guided cells to neuronal differentiation, and a neuronal network was established within a short time [94].

In addition to scaffolds, nanoparticles have shown beneficial effects in cell therapy for NDs. For example, Marcuzzo et al. [95] administrated gold nanoparticles loaded with FM19G11, a hypoxia-inducible factor modulator, to ependymal stem progenitor cells isolated from G93A-SOD1 mice. A significant impact on cell regeneration and proliferation was observed, indicating a therapeutic potential to delay ALS progression [95]. 

### 5.2. In Vivo Studies

The combined use of biomaterials and stem cells shows superior results in animal models of neurological pathologies or vascular events. The nature of the biomaterials used has been variable, from organic or synthetic compounds. For example, the use of carbon nanotubes improves the survival and neuronal differentiation of NSCs from the human olfactory bulb, after implantation in rats with TMT-induced neurodegeneration, restoring cognitive deficits and neurodegenerative changes; therefore, this biomaterial is a strong candidate as a therapeutic vehicle for these cells for the treatment of various NDs [96]. 

In the study of Alzheimer’s disease, using animal models, one of the strategies used has been prior in vitro induction with copolymeric nanoparticles of poly(ethylene glycol) and polylactic-co-glycolic acid loaded with nerve growth factor, facilitating the differentiation of NSC in vitro and may also improve functional recovery in vivo, compared to NSC therapy without the nanoparticles [97].

In mouse models of PD, scaffolds composed of short PLLA nanofibers embedded with a thermosensitive xyloglucan hydrogel and loaded with glial-derived neurotrophic factor (GDNF) have been used to deliver ventral midbrain dopaminergic progenitor cells [98]. The scaffold did not have a negative impact on the host response and increased cell survival and reinnervation of the striatum [98]. Another research group used in situ polymerization of a fibrin scaffold to engraft MSCs that overexpress GDNF; the biomaterial did not show adverse effects related to cell survival or GDNF release. The use of the scaffold was also associated with the modulation of reduced host microglial and astroglial response, which enhanced the therapeutic properties of these cells in adult rats [99]. In a more recent study, in 2019, Gómez-Pinedo et al. [100] used scaffolds loaded with permissive olfactory sheathing glia to bridge the substantia nigra and striatum, promoting colonization and migration of TH+ axons between both ends of the graft.

In a mouse model of multiple sclerosis (EAE), Hoveizi et al. [101] administered a PLLA/chitosan scaffold preloaded with NLC derived from PC12 cells, observing reductions in axonal damage and severity of demyelination, and an improvement in the clinical course of EAE. In vitro, the scaffolds promoted neuronal cell adhesion, growth, and differentiation. Similarly, Matías-Guiu et al. [102] proposed chitosan and its derivatives as the most appropriate biomaterials to design OPC-containing particles to be administered through the nose-to-brain route as an alternative treatment for MS or other demyelinating pathologies. Among the most relevant characteristics of chitosan, the authors mention its ability to promote axonal regeneration, its anti-inflammatory properties, and its ability to successfully deliver neurotrophic factors and cells, leading to functional recovery in various models [102].

In recent years, promising results of biomaterials as structural support in CVD have been published. For example, hyaluronic acid scaffolds with and without adipose stem cells have been implanted at the site of injury in a model of focal stroke. The biomaterial favored proliferation, neurogenesis, and capillarization at the site of injury; this effect was enhanced by the addition of stem cells [103].

## 6. Future Perspectives

To our knowledge, no specific research has been conducted to date into the joint use of biomaterials and intranasal cell therapy. In the light of the promising results reported for the joint use of biomaterials and cell therapy and for nose-to-brain administration, we believe that this will be the next major line of research which will enable effective and non-invasive administration of stem cells for the treatment of NDs and CVD. However, we are far from fully understanding the connection between the characteristics of biomaterials and their effects on cells. An important relationship is known to exist between the mechanical properties of the biomaterial to be used and the tissue we aim to repair. However, biomaterials must be specifically designed for each disease due to changes to the extracellular matrix during the progression of NDs; for example, the greater softness of the tissue in such diseases as AD and MS [104]. In addition to their role in the diseases addressed in this review, the treatment strategies discussed may also be of interest in the context of epilepsy [105] and such oncological conditions as glioblastoma multiforme [106]. However, even with the assistance of biomaterials, intranasal cell therapy presents some limitations, such as problems with the biodistribution of cells in the brain. Zhang et al. [58] discuss how the biodistribution of intranasally administered cells is affected by the animal model used and the number of cells administered; furthermore, tracing cells can be a challenging task. The authors stress the importance of certain secretory substances that may selectively guide cell migration, such as extracellular vesicles, which are known to participate in the crosstalk communication between cells and tissue repair processes [107]. These may be the focus of future studies into nose-to-brain cell therapy and cell-free therapy. Studies into other CNS disorders, such as traumatic brain injury, have shown that the concomitant use of biomaterials and genetic engineering of stem cells may increase treatment effectiveness [108]; this may be an interesting area of research in the context of NDs. Finally, biomaterials may play an important role in preventing or reducing the immune response to the transplantation of non-autologous cells, mimicking the host tissue using materials that are cationic and biocompatible with the nervous tissue, facilitating the integration of the engrafted cells [102].

## Figures and Tables

**Table 1:** Cell Types Used In The Intranasal Route.

Cell Type	Advantages	Limitations
**MSCs**	High accessibility from adipose tissue, bone marrow, umbilical cord, and perinatal derivatives Easy isolation Low immunogenicity Differentiation into neurons and glial cells Suppress the inflammation Prevent oxidative stress Promote neuroprotection and remyelination Secretion of neurotrophic factors Modulation of immune system reducing macrophage infiltration and microglia activation Promote angiogenesis	Invasive procedures and limited number of cells Risk of tumor formation
**NSCs**	Differentiation into neurons, astrocytes, and oligodendrocytes Secretion of neurotrophic factors Promote angiogenesis and neurogenesis Decrease microglia response Improve myelination	Ethical issues for obtention Limited differentiation Invasive procedures and limited number of cells Limited proliferation and expansion Limited availability Difficult isolation
**iPSCs**	Low risk of immune rejection Differentiation into different cell types in vitro by different reprogramming methods No ethical issues because avoids the use of fetus	Cells are not identical to embryonic ones Risk of tumor formation Risk of insertional mutagenesis in the reprogramming process Low efficacy of reprogramming process

## Data Availability

Not applicable.

## Supplementary Materials

The following supporting information can be downloaded at: https://www.mdpi.com/article/10.3390/cells11193095/s1, Table S1: Intranasal cell therapy clinical trials (clinicaltrials.gov).
