# Supplementary material for: Nose-to-Brain: The Next Step for Stem Cell and Biomaterial Therapy in Neurological Disorders
Source: Cells. 2022 Oct 1;11(19):3095. doi: 10.3390/cells11193095 (PMC9564248; doi:10.3390/cells11193095)
Supplement: Supplementary file 1 [file cells-11-03095-s001.zip › cells-1919382-supplementary.pdf]

Table S1. Nose-to-brain cell therapy in neurological disorders

Neurological disorder	Animal model	Cell type	Administered cells per animal	Markers	Results	Reference (Year)
**Alzheimer's disease**	APP/PS1	Macrophages	1,5 x 10^6	eGFP	• Cells reach the hippocampus and occipital cortex<br>• Cells accumulate near the Aβ plaques	Danielyan et al. (2014) [16]
	APP/PS1	hNSCs	2 x 10^6	Stem121	• Decrease in Aβ plaques in hippocampus and cortex<br>• Differentiation to cholinergic neurons<br>• Possible increase in neurogenesis<br>• Improvement in learning and memory	Lu et al. (2021) [17]
**Parkinson's disease**	6-OHDA	MSCs	3 x 10^5<br>5 x 10^5	EGFP	• Survival after 4.5 and 6.7 months<br>• Increase in TH and dopamine in the corpus striatum and substantia nigra<br>• Locomotion improvement	Danielyan et al. (2011) [23]
	6-OHDA	hMSCs	2 x 10^6	NIR815 + Hoechst 33258	• Infrared signal in olfactory epithelium 10 min after administration which disappeared within an hour<br>• No signal was detected in the brain	Bossolasco et al. (2012) [24]
	(Thy)-h[A30P] αS	MSCs	1 x 10^6	eGFP	• Cells were observed in olfactory bulbs and brainstem near phosphorylated α-synuclein aggregates	Danielyan et al. (2014) [16]

	MPTP	BM-NSCs	3 x 10^5	GFP	• Less dopaminergic neurons loss<br>• Decrease in inflammation<br>• Behavioural improvement	Li et al. (2016) [25]
	Rotenone	MSCs	5 x 10^5	MPIO particles	• Increase in dopaminergic neurons<br>• Locomotion improvement	Salama et al. (2017) [27]
	MPTP	DPSCs	5 x 10^5	PKH-26	• Increase in TH 4 weeks after administration<br>• Improvement in sensorimotor coordination	Simon et al. (2019) [26]
	6-OHDA	HEDSCs	1 x 10^4<br>**5 x 10^4**<br>1 x 10^5	GFP	• Increase in dopaminergic neurons in substantia nigra pars compacta<br>• Behavioural improvement	Bagheri-Mohammadi et al. (2019) [28]
	6-OHDA	OE-MSCs	Not specified	None	• Increase in dopaminergic markers<br>• Behavioural improvement	Alizadeh et al. (2021) [29]
**Huntington's disease**	R6/2	MSCs	2 x 10^6	eGFP	• Decrease of inflammation in olfactory bulbs, hippocampus, and corpus striatum<br>• Increase of dopaminergic and medium spiny neuron markers<br>• Survival after 11 weeks<br>• Improvement in motor behaviour	Yu-Taeger et al. (2019) [31]
	EAE	aNSCs	1 x 10^6	GFP	• Cells reach the olfactory bulb, cortex, hippocampus, corpus striatum, brainstem, and spinal cord in 7 days	Wu et al. (2013) [37]

**Multiple sclerosis**					• High number of cells in demyelinating areas in 21 days<br>• Anti-inflammatory effect in the CNS<br>• Remyelination increases in the ventral column and spinal cord<br>• Stem cell differentiation towards mature and NG2+ oligodendrocytes, astrocytes, and neurons	
	EAE	MSCs (genetically modified)	5 x 10^6	GFP HuNu	• Cells reach the olfactory bulb, entorhinal cortex, and cerebellum<br>• Inflammation and demyelination decrease	Fransson et al. (2014) [36]
**Ischaemic stroke**	Permanent occlusion of MCA	BMSCs	1 x 10^6	Hoechst 33342	• Cells reach the CNS in 1.5 hours<br>• Preconditioned cells gather in ischaemic areas<br>• Reduction in the infarct volume<br>• Less cellular death<br>• Improvement in sensorimotor coordination	Wei et al. (2013) [50]
	Permanent occlusion of MCA	BMSCs	1 x 10^6	Hoechst 33342	• Indicative markers of neurogenesis and angiogenesis<br>• Better local cerebral blood flow<br>• Improvement in motor coordination	Shen et al. (2021) [51]
**Neonatal hypoxic-ischaemic brain injury**	Unilateral carotid artery occlusion and hypoxia	MSCs	0, 25 x 10^6<br>0,5 x 10^6<br>1 x 10^6	PKH-26	• Less grey and white matter loss<br>• Cells reach lesion areas and spread to adjacent regions<br>• Improvement in sensorimotor coordination	Donega et al. (2013) [44]

	Unilateral carotid artery occlusion and hypoxia	MSCs	0,5 x 10^6	None	• Beneficial effects (anterior row) remain for 14 months post-injury<br>• Neither tumour nor malformations were detected in nasal turbinates, brain, and other organs	Donega et al. (2015) [45]
	Ligation of distal branches of the MCA and permanent cauterization of the common carotid artery	BMSCs	1 x 10^6	None	• Reduction in the infarct volume<br>• Less BBB disruption<br>• Indicative markers of neurogenesis and angiogenesis<br>• Better local cerebral blood flow<br>• Improvement in sensorimotor, olfactory, and social functions	Wei et al. (2015) [46]

Animal model: MCA (middle cerebral artery) Cells: hNSCs (human neural stem cells), BM-NSCs (bone marrow-derived neural stem cells), aNSCs (adult neural stem cells), MSCs (mesenchymal stem cells), OE-MSCs (human olfactory ectomesenchymal stem cells), DPSCs (dental pulp stem cells), HEDSCs (human endometrium-derived stem cells). Markers: GFP (green fluorescent protein), eGFP (enhanced green fluorescent protein), MPIO (micrometre-sized iron oxide), HuNu (Human Nuclei). Other words: HI (hypoxic-ischemic), IR (infrared).
